# Compost mediates the recruitment of core bacterial communities in alfalfa roots to enhance their productivity potential in saline-sodic soils

**DOI:** 10.3389/fmicb.2024.1502536

**Published:** 2024-11-22

**Authors:** Tian-Jiao Wei, Guang Li, Yan-Ru Cui, Jiao Xie, Xing Teng, Yan-Jing Wang, Zhong-He Li, Fa-Chun Guan, Zheng-Wei Liang

**Affiliations:** ^1^Jilin Academy of Agricultural Sciences, China Agricultural Science and Technology Northeast Innovation Center, Changchun, China; ^2^Northeast Institute of Geography and Agroecology, Chinese Academy of Sciences, Changchun, China

**Keywords:** compost, saline-sodic soil, core rhizosphere bacterial community, alfalfa, plant productivity

## Abstract

**Introduction:**

Composting is one of the effective environmental protection and sustainable measures for improving soil quality and increasing crop yield. However, due to the special physical and chemical properties of saline-sodic soil and the complex rhizosphere microecological environment, the potential mechanism of regulating plant growth after applying compost in saline-sodic soil remains elusive.

**Methods:**

Here, we investigated the effects of different compost addition rates (0, 5, 15, 25%) on plant growth traits, soil chemical properties, and rhizosphere bacterial community structure.

**Results:**

The results showed that compost promoted the accumulation of plant biomass and root growth, increased soil nutrients, and enhanced the diversity and complexity of the rhizosphere bacterial communities. Moreover, the enriched core bacterial ASVs (Amplicon Sequence Variants) in compost treatment could be reshaped, mainly including dominant genera, such as *Pseudomonas*, *Devosia*, *Novosphingobium*, *Flavobacterium*, and *Allorhizobium-Neorhizobium-Pararhizobium-Rhizobium*. The functions of these ASVs were energy resources and nitrogen cycle functions, suggesting the roles of these ASVs in improving plant root nutrient resource acquisition for alfalfa growth. The contents of available potassium, available phosphorus, total nitrogen, and organic carbon of the soil surrounding the roots, the root length, root surface area, root volume, and root tips affected the abundance of the core bacterial ASVs, and the soil chemical properties contributed more to the effect of plant biomass.

**Discussion:**

Overall, our study strengthens the understanding of the potentially important taxa structure and function of plant rhizosphere bacteria communities, and provides an important reference for developing agricultural microbiome engineering techniques to improve root nutrient uptake and increase plant productivity in saline-sodic soils.

## 1 Introduction

Globally, soil salinization is one of the most critical issues threatening agricultural production, food security, and sustainability in arid and semi-arid regions (Julkowska and Testerink, 2015). The western Songnen Plain stands as one of the world’s three major regions with concentrated saline-affected soil, covering 3.78 million hectares of saline-sodic soil. This soil is primarily marked by NaHCO_3_ and Na_2_CO_3_, inducing oxidative and osmotic stress on plants, ultimately impairing plant growth and disrupting nutrient equilibrium (Wei et al., 2020; Yang et al., 2016; Wei et al., 2021). Therefore, it is necessary to find an effective, environmentally friendly, and sustainable ecological restoration strategy to maintain crop production and restore soil health.

The improper use of chemical fertilizers has posed a serious threat to the agricultural ecosystem, leading to reduced microbial diversity, loss of soil organic carbon, and disruption of nitrogen cycling (Feng et al., 2018; Harindintwali et al., 2021). Compost is the decomposition of a variety of organic waste into stable, harmless organic fertilizer products suitable for soil fertilizer, it can also reduce the waste of agricultural residues and animal manure (Alromian, 2020). Compared with chemical fertilizer, the nutrient content in compost is rich, the release is slower and durable, and it can better supply the needs of plant growth and development. Replacing part of chemical fertilizer with compost can increase yield, increase nitrogen recovery rate, promote the propagation of soil microorganisms, and improve soil biological characteristics, which is conducive to improving soil fertility (Cao et al., 2020). The application of compost will also improve soil porosity, increase soil aggregate stability, regulate plant nutrient balance, and ultimately regulate overall plant growth and yield (Li et al., 2021; Sharma et al., 2021). Composting is considered one of the best ways to regenerate soil organic carbon in farmland, reduce the need for mineral fertilizers, increase crop yields, and achieve agricultural recycling and sustainability.

The rhizosphere is described as a dynamic niche where soil microorganisms interact with plant roots (Bais et al., 2006). The interaction between rhizosphere microorganisms and plants can affect the material circulation and energy flow, and the changes in community structure and abundance can affect the growth and development of plants (Wagg et al., 2011). Microorganisms in the rhizosphere are recruited by root exudates, primarily through nutrient acquisition, pathogen resistance, and hormone secretion, often associated with the growth and health of their hosts (Berendsen et al., 2012). Core microbial groups are prevalent in different environments and drive multiple ecosystem functions (Shade and Handelsman, 2012; Durán et al., 2018; Chang et al., 2022). The introduction of external fertilizers has the potential to influence the functional microbiome of the soil and its interaction with ecosystem diversity. Soil nutrient availability plays a direct role in regulating the majority of functional microbial communities within the soil (Hu et al., 2024). Although there is evidence that the addition of exogenous fertilizers can enhance the carbon sequestration of saline-alkali soils and increase the stability and complexity of bacterial communities (Jiang et al., 2024). However, the relationship among core rhizosphere bacterial communities, soil nutrients, and their contributions to plant productivity potential remain elusive.

Alfalfa (*Medicago sativa* L.) is a leading feed choice due to its strong salt tolerance, high yield and excellent quality. Additionally, its extensive root system creates additional ecological niches for rhizosphere microorganisms (Ju et al., 2020). Alfalfa is widely cultivated in the north of China, but the saline-sodic soil in the west of Songnen Plain would seriously inhibit the growth of alfalfa and reduce the yield (Wei et al., 2020). Although recent studies have documented compost as a soil modification applied in crops and vegetables, such as corn (Li et al., 2022), sorghum (Yin et al., 2022), radish (Xu et al., 2023), and mustard (Huang et al., 2010). Nevertheless, the information concerning on the potential mechanism of regulating plant growth after applying compost is poor. Here, we aimed to (1) identify the most suitable compost application rate for the reclamation of saline-sodic soil; (2) characterize the compost application on the growth characteristics of alfalfa, the properties of saline-sodic soil, and the diversity and structure of rhizosphere bacterium communities; (3) to elucidate the potential contribution of core bacterial communities to soil nutrients and plant growth under different compost application rates. This study enhanced our understanding of the structure and function of composting to repair saline-alkali soil bacterial communities and also provided a new design idea for the development of saline-alkali soil microbial joint repair strategies.

## 2 Materials and methods

### 2.1 Soil, compost and pot experiment

The saline-sodic soil was collected (0–20 cm) from the Da’an Sodic Land Experimental Station (45°36′ N, 123°53′ E, and 132.1 m) in the Songnen Plain, northeast China. The compost product used in this study is provided by Zhongsheng Environmental Protection Technology Development Co., Ltd. (Changchun, Jilin, China). Compost product was mainly fermented from corn stalks and chicken manure. The chemical properties of the soil and compost are shown in Supplementary Table S1.

Alfalfa (*Medicago sativa* L. “Gongnong NO. 1”) seeds were sterilized with a 0.6% sodium hypochlorite solution for 10 min, rinsed with distilled water three times, and then germinated in the plastic pots (15.6-cm diameter × 15.5-cm depth). We selected the cultivar “Gongnong NO.1” due to its tolerance to saline-alkaline stress, as determined in our prior study (Wei et al., 2020, 2021). The experiment was based on a composition of compost and soil matrix blended in a certain mass proportion by Liu et al. (2019) and Mazumder et al. (2021). Specifically, as indicated in Supplementary Table S2, four treatments-named SA, SAF5, SAF15, and SAF25-varying in the rate of compost application were applied, together with the compost and saline-sodic soil at thresholds of 0:100, 5:95, 15:85, and 25:75. The control soil (SA) is saline-alkali soil with 0% compost added and no other fertilizers added. A controlled growth chamber was set to 25°C day /20°C night with a 12-h photoperiod at 350 μmol photons m^−2^ s^−1^ light intensity.

The experimental design is based on our previous research, all of the experiments were conducted with three biological replicates, each treatment consisting of three pots of alfalfa seedlings, with 15 seedlings in each pot. The pots were rotated within the growth chamber every 1–2 days to minimize any effect of location (Wei et al., 2021). After seedings were planted, water (EC 1.05 mS/cm, pH 7.52) was used for irrigation. During the growth period, all potted plants were adjusted to 60% field water capacity with tap water, and there was no additional fertilization for all treatments.

### 2.2 Sample collection and processing

The rhizosphere soil was collected after 8 weeks of cultivation according to the method of Chang et al. (2022). Briefly, the root was immersed in a tube filled with 5 mL of sterile water, and 1 mm of soil around the roots was collected. The centrifuge tube was centrifuged for 30 s at a relative centrifugal force of 10,000 × g. After the removal of the supernatant, rhizosphere soil samples were used for microbial sequencing. In addition, the loose soil around the roots was collected and air-dried and through a 2 mm sieve to determine the chemical properties of the soil.

### 2.3 Measurement of plant growth and soil properties

Five seedlings in each treatment were scanned using an Epson Expression 10000XL root scanner (Epson America Inc., Long Beach, CA, United States). Then WinRHIZO root analysis software (Regent Instruments Canada Inc., Ville de Québec, QC, Canada) was used to analyze root morphology parameters, including root length (RL), root surface area (RS), root volume (RV), and root tips (RT).

Then, the five seedlings under each treatment were taken as a group and cut off from the root, and divided into shoots and roots. Subsequently, these samples were placed in the oven (DHG101S, Subo Corporation, Shaoxing, China), defoliated at 105°C for 15 min, then dried to the constant weight at 75°C, and the dry weight of the shoot (SDW)/root (RDW) per group was weighed, and then the SDW and RDW of per plant were calculated using the corresponding values divide by 5. The total biomass (TDW) was calculated by adding the corresponding values. The soil pH, the contents of total nitrogen (TN), total phosphorus (TP), total potassium (TK), available nitrogen (AN), available phosphorus (AP), available potassium (AK), and soil organic carbon (SOC) in soil were determined as described by Wang et al. (2018).

### 2.4 DNA extraction and sequencing

Total microbial community DNA was isolated from 0.25 g of soil per sample using the FastDNA® SPIN Kit For soil Kit (MOBIO Laboratories Inc., CA, United States). NanoDrop spectrophotometer (ND2000, Thermo Scientific, DE, United States) and agarose gel electrophoresis to assess genomic DNA concentration and integrity. Bacterial 16 s rDNA gene was amplified with 338F (5′-ACTCCTACGGGAGGCAGCA-3′) and 806R (5′-GGACTACHVGGGTWTCTAAT-3′) universal primers of bacterial 16 s rDNA gene V3-V4. The sequencing was performed on the Illlumina NovaSeq platform and 250 bp paired-end reads were generated. The 16S sequences were uploaded to National Centre for Biotechnology Information (NCBI) Sequence Read Archive (SRA) under the accession numbers PRJNA1133892.

### 2.5 Bioinformatics analysis and statistical analysis

Microbiome bioinformatics were performed with QIIME2 2019.4 (Bolyen et al., 2018) with slight modification according to the official tutorials.^1^ Briefly, raw sequence data were demultiplexed using the demux plugin following by primers cutting with cutadapt plugin. Sequences were then quality filtered, denoised, merged and chimera removed using the DADA2 plugin. After quality control, ASVs (amplicon sequence variants) were compared with template sequences in fasttree2 database to obtain species classification information. ASV-level *α*-diversity indices, such as Chao1 richness estimator, Faith’s PD, Observed species, Good’s coverage, Pielou’s evenness, Shannon diversity index, and Simpson index were calculated using the ASV table in QIIME2. ASV-level *β*-diversity analysis was performed to investigate the structural variation of microbial communities across samples using Jaccard metrics. Bray-Curtis metrics and UniFrac distance metrics and visualized via principal coordinate analysis (PCoA). The redundancy analysis (RDA) of environmental variables and microbial community structure was carried out using the vegan package in R software. The constructed for the rhizosphere communities network by R package *microeco* and used to visualize network diagrams with Gephi 0.10.1 software (Liu et al., 2020). The core ASVs in soil were defined as the occurrence frequency higher than 80% of samples and average relative abundance higher than 0.1% (Durán et al., 2018). The volcano plot of the average abundance of core ASVs for different compost application rates compared to SA was obtained by using the DESeq2 package in R software (Love et al., 2014). The differentially abundant ASV with the parameters of false discovery rate (FDR) below 0.05 and absolute log_2_ (fold change) ≥ 1 were considered different significant ASVs. The shared and particularly enriched and depleted ASVs in different compost treatments were plotted in Venn diagram. The ecological functions of enriched core ASVs were predicted by using FAPROTAX software (Sansupa et al., 2021). The heatmap function of enriched core ASVs was conducted using ComplexHeatmap package on the R platform (Gu, 2022). The prediction of alfalfa biomass by bacterial genus and soil chemical properties in different compost treatments was conducted using the randomForest package on the R platform (Breiman, 2001; Shen et al., 2020). Microsoft Excel 2016 software was used to organize the test data. The data were analyzed using one-way ANOVA, followed by Duncan’s multiple range tests. *p*-values < 0.05 were considered as statistically significant.

## 3 Results

### 3.1 Plant traits and soil properties

**Figure 1:**
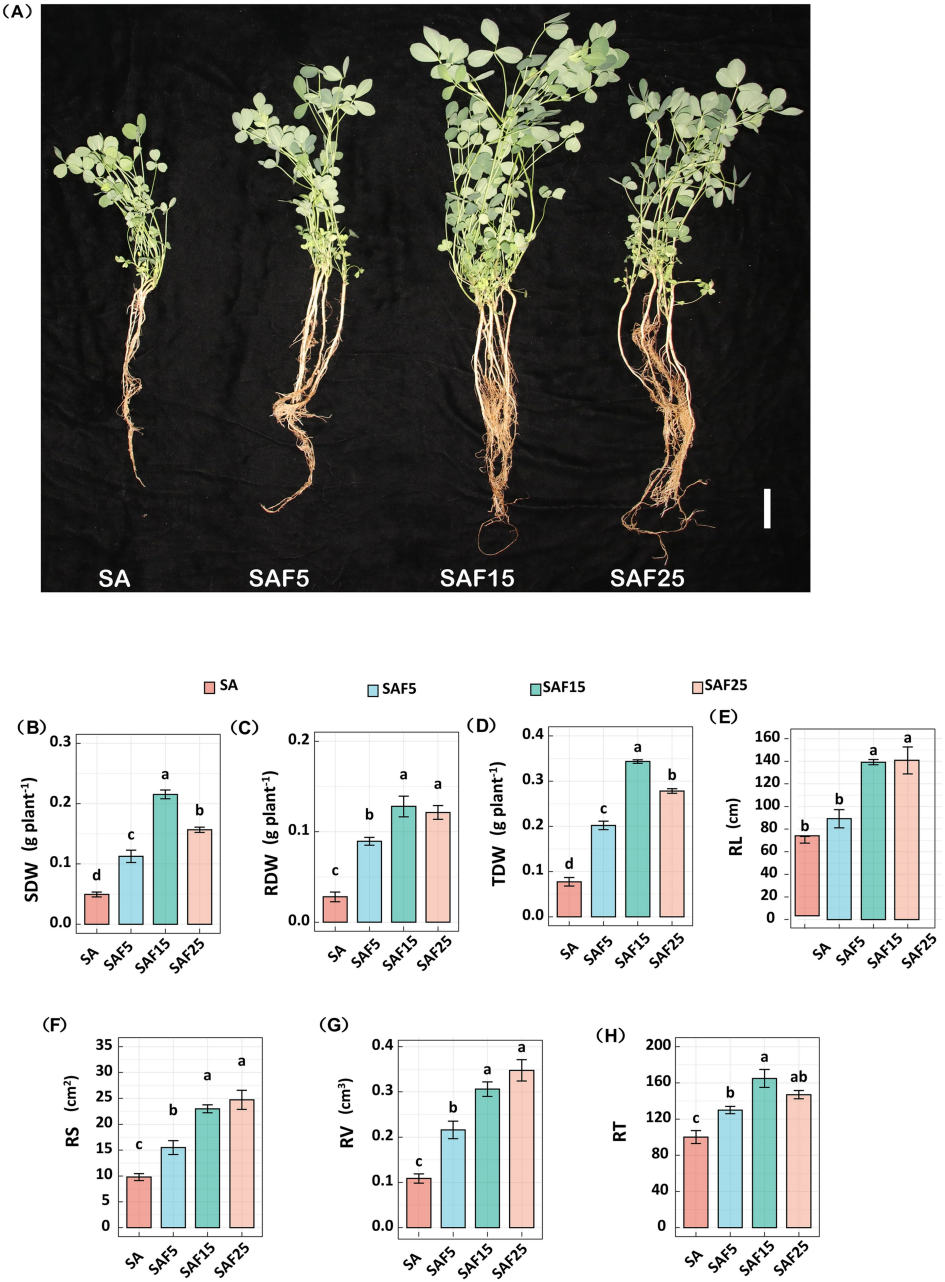
Effects of compost on biomass and root morphological parameters of alfalfa seedlings. (A) Photographs of seedling growth were taken after alkaline treatment for 8 weeks. Bar = 5 cm. The SDW (B) RDW, (C), TDW (D) of alfalfa seedlings under different compost application rates. The RL (E), RS (F), RV (G), and RT (H) of alfalfa seedlings under different compost application rates. All values are shown as mean ± standard error. Different lowercase letters indicate significant differences among compost treatments at *p* < 0.05 by Duncan’s multiple range test. The SA, SAF5, SAF15, and SAF25 represent different compost application rates, respectively. SDW: the dry weight of shoot; RDW: the dry weight of root; TDW: the dry weight of total plant; RL: root length; RS: root surface area; RV: root volume; RT: root tips.

Compost application significantly affected the growth of alfalfa seedlings (Figure 1A). After compost application, the SDW of alfalfa seedlings significantly increased by 128.38, 336.49, and 217.57%, respectively (*p* < 0.05) (Figure 1B). The RDW of alfalfa seedlings in SAF15 treatment was the highest, which was 357.14% higher than that in SA treatment (*p* < 0.05). Meanwhile, the RDW of alfalfa seedlings in SAF15 and SAF25 treatments were 43.28 and 35.82% higher than that in SAF5, respectively (*p* < 0.05), but there was no significant difference between the two treatments (*p* > 0.05) (Figure 1C). The TDW of alfalfa seedlings in SAF5, SAF15, and SAF25 treatments were 2.61-fold, 4.44-fold and 3.59-fold of that in SA treatment, respectively (Figure 1D).

The application of compost had a significant effect on the root morphology parameters of alfalfa seedlings. Compared with SA treatment, the RL of alfalfa seedlings in SAF5, SAF15, and SAF25 treatments significantly increased by 26.37, 97.24, and 99.45%, respectively (*p* < 0.05) (Figure 1E). The RL of alfalfa seedlings in SAF15 and SAF25 treatments were 56.08 and 57.83% higher than that in SAF5 treatments (*p* < 0.05), but there was no significant difference between SAF15 and SAF25 treatments (*p* > 0.05). Compared with SA treatment, the RS of alfalfa seedlings in SAF5, SAF15, and SAF25 treatments were significantly increased by 58.26, 135.16, and 152.90%, respectively (*p* < 0.05). The RS of alfalfa seedlings in SAF15 and SAF25 treatment were 48.59 and 59.80% higher than that under SAF5 treatment, and there was no significant between them (Figure 1F). Compared with SA treatment, the RV of alfalfa seedlings in SAF5, SAF15, and SAF25 treatments were significantly increased by 99.26, 182.29, and 220.73%, respectively (*p* < 0.05) (Figure 1G). The RV of alfalfa seedlings in SAF15 treatment and SAF25 treatment were 1.42-fold and 1.61-fold of that in SAF5 treatment, respectively, and this difference between them was not significant. Compared with SA treatment, the RT of alfalfa seedlings in SAF5, SAF15, and SAF25 treatments were significantly increased by 29.85, 64.76, and 46.84%, respectively (Figure 1H) (*p* < 0.05). The RT of alfalfa seedlings in SAF15 treatment was the highest, which was 1.27-fold of SAF5 treatment and 1.12-fold of SAF25 treatment.

The addition of compost changes soil pH and soil nutrients. The contents of AN, AP, AK, TN, TP, and SOC (Table 1) under different compost application rates treatments were significantly higher than those in SA treatment (*p* < 0.05). In contrast, the soil pH was significantly lower than that in SA treatment (*p* < 0.05) (Table 1). Meanwhile, the contents of AP, AK, TN, TP, and SOC increased substantially with the increase in compost application rates and reached the maximum in SAF25 treatment. The contents of AN and TK in SAF15 and SAF25 treatments were significantly higher than those in SAF5 (*p* < 0.05), but there was no significant difference between them (*p* > 0.05).

**Table 1:** Differences in soil properties surrounding the roots of alfalfa seedlings under different compost application rates.

Treatment	pH	AN (mg kg^−1^)	AP (mg kg^−1^)	AK (mg kg^−1^)	TN (g kg^−1^)	TP (g kg^−1^)	TK (g kg^−1^)	SOC (g kg^−1^)
SA	9.12 ± 0.04a	59.05 ± 2.37a	30.95 ± 0.70d	93.68 ± 2.62c	0.37 ± 0.01d	0.41 ± 0.00d	25.93 ± 0.20a	2.94 ± 0.07d
SAF5	8.93 ± 0.01b	75.11 ± 4.24a	102.74 ± 1.10c	183.38 ± 6.53c	0.86 ± 0.02c	0.66 ± 0.02c	23.42 ± 0.51a	8.84 ± 0.07c
SAF15	8.48 ± 0.01c	212.90 ± 6.47b	148.53 ± 2.62b	465.58 ± 9.42b	2.13 ± 0.09^b^	1.18 ± 0.05b	23.19 ± 1.27ab	18.58 ± 0.14^b^
SAF25	8.04 ± 0.01d	213.42 ± 8.48b	188.35 ± 1.96a	1001.72 ± 71.56a	4.29 ± 0.17a	1.74 ± 0.08a	20.18 ± 01.27b	29.30 ± 0.29a

All values are shown as mean ± standard error. Different lowercase letters indicate significant differences among compost treatments at *p* < 0.05 by Duncan’s multiple range test. AN, available nitrogen; AP, available phosphorus; AK, available potassium; TN, total nitrogen; AK, available potassium; TP, total phosphorus; TK, total potassium; SOC, soil organic carbon. The SA, SAF5, SAF15, and SAF25 represent different compost application rates, respectively.

### 3.2 Rhizosphere bacterial community diversity and co-occurrence networks

**Figure 2:**
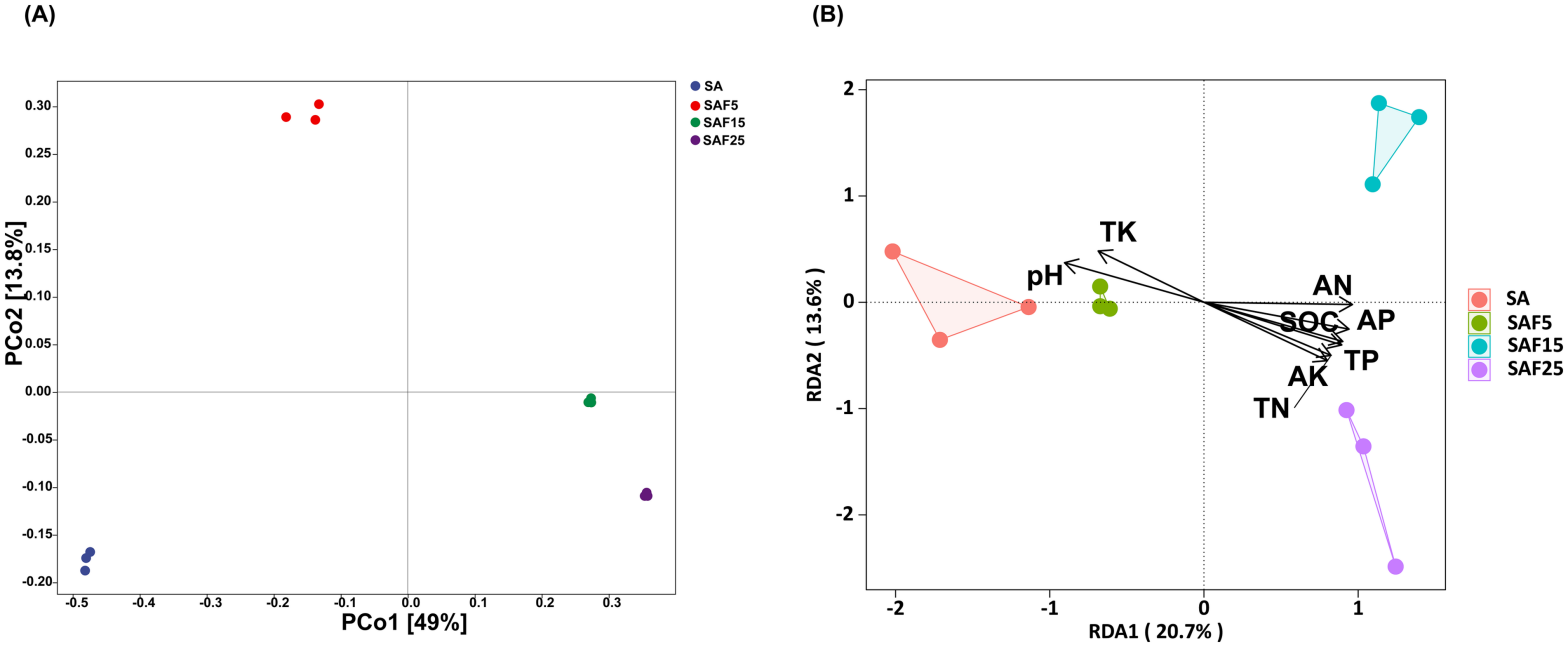
Principal coordinate analysis (PCoA) based on Bray-Curtis distance, with 95% ellipse confidence under different compost application rates (A). Redundancy analysis (RDA) of the relationship between the soil properties and rhizosphere bacterial communities (B). The SA, SAF5, SAF15, and SAF25 represent different compost application rates, respectively.

The PCoA analysis indicated the close distance between SAF15 and SAF25 treatments, the distance between SAF5, SAF15, and SAF25 treatments and SA treatments is longer. At the same time, the soil bacterial communities under low compost addition rate were not similar to that under medium and high compost addition rates. The cumulative contribution of X axis (49%) and Y axis (13.8%) reached 62.8%, indicating that compost could significantly change the microbial composition of saline-sodic soil (Figure 2A). The RDA analysis showed the eigenvalues of the first and second axes were 20.7 and 13.6%, respectively. The soil pH, AN, AP, and SOC were the main environmental factors affecting the structure of rhizosphere bacterial communities (Figure 2B).

Compared with SA treatment, the bacterial diversity index of Chao1, Faith_pd and observed_species decreases first and then increases significantly with the increase of compost application amount, especially reaching the maximum value under SAF15 treatment (Table 2). The bacterial diversity index of Pielou_e, Shannon and Simpson all showed a decreasing trend with the increase of compost application amount (Table 2).

**Table 2:** Differences in the α-diversity of soil bacterial communities under different compost application rates.

Treatment	Chao1	Faith_pd	Observed_species	Goods_coverage	Pielou_e	Shannon	Simpson
SA	2,194 ± 413bc	150 ± 20b	2,168 ± 403b	0.9983 ± 0.0007a	0.9168 ± 0.0043a	10.10 ± 0.24a	0.9978 ± 0.0003a
SAF5	1,960 ± 155b	141 ± 10b	1,944 ± 152b	0.9989 ± 0.0003a	0.9002 ± 0.0087b	9.82 ± 0.07a	0.9965 ± 0.0005b
SAF15	3,199 ± 99a	195 ± 3a	3,018 ± 75a	0.9889 ± 0.0010b	0.8624 ± 0.0013c	9.97 ± 0.02a	0.9974 ± 0.0001ab
SAF25	2,838 ± 181ab	194 ± 5a	2,655 ± 129ab	0.9897 ± 0.0019b	0.8150 ± 0.0026d	9.27 ± 0.03b	0.9926 ± 0.0001c

All values are shown as mean ± standard error. Different lowercase letters indicate significant differences among compost treatments at *p* < 0.05 by Duncan’s multiple range test. The SA, SAF5, SAF15, and SAF25 represent different compost application rates, respectively.

**Figure 3:**
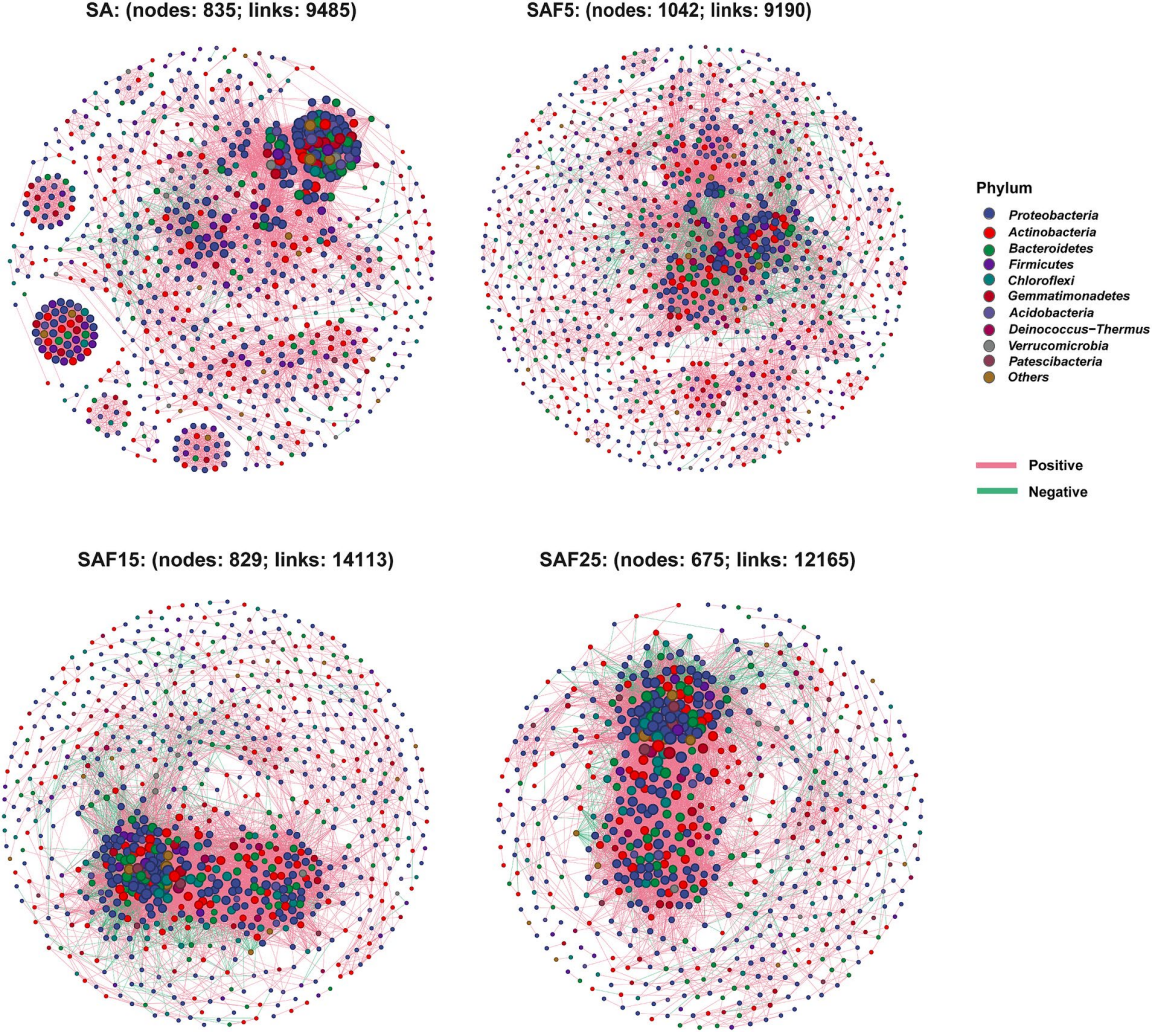
Co-occurring network of bacterial communities at the phylum level under different compost application rates. The SA, SAF5, SAF15, and SAF25 treated networks were constructed using the relative abundance of phylum with average relative abundance >1%. Nodes indicate the phylum involved in the networks, and edges indicate the relationships among the nodes. Red lines represent positive (Spearman’s correlation, *r* ≥ 0.7 and *p* ≤ 0.01) relationships and green lines represent negative (Spearman’s correlation, *r* ≤ −0.7 and *p* ≤ 0.01) relationships. The different colored dots represent the different phylum to which the genera belong. The SA, SAF5, SAF15, and SAF25 represent different compost application rates, respectively.

Based on the abundance data of microbial communities at the phylum level, the relationships and interactions among different microbial groups were studied using Spearman’s rank symbiotic network analysis. Compared with SA treatment, the number of total edges significantly increased under compost application rates. The number of total edges in SAF15 treatment was the highest, which was 1.49-fold that of the SA treatment, followed by SAF5 and SAF25. In addition, the number of nodes of SAF5 was the largest, followed by SA, SAF15, and SAF25 (Figure 3; Supplementary Table S3).

### 3.3 The composition and function of the core rhizosphere bacterial community

**Figure 4:**
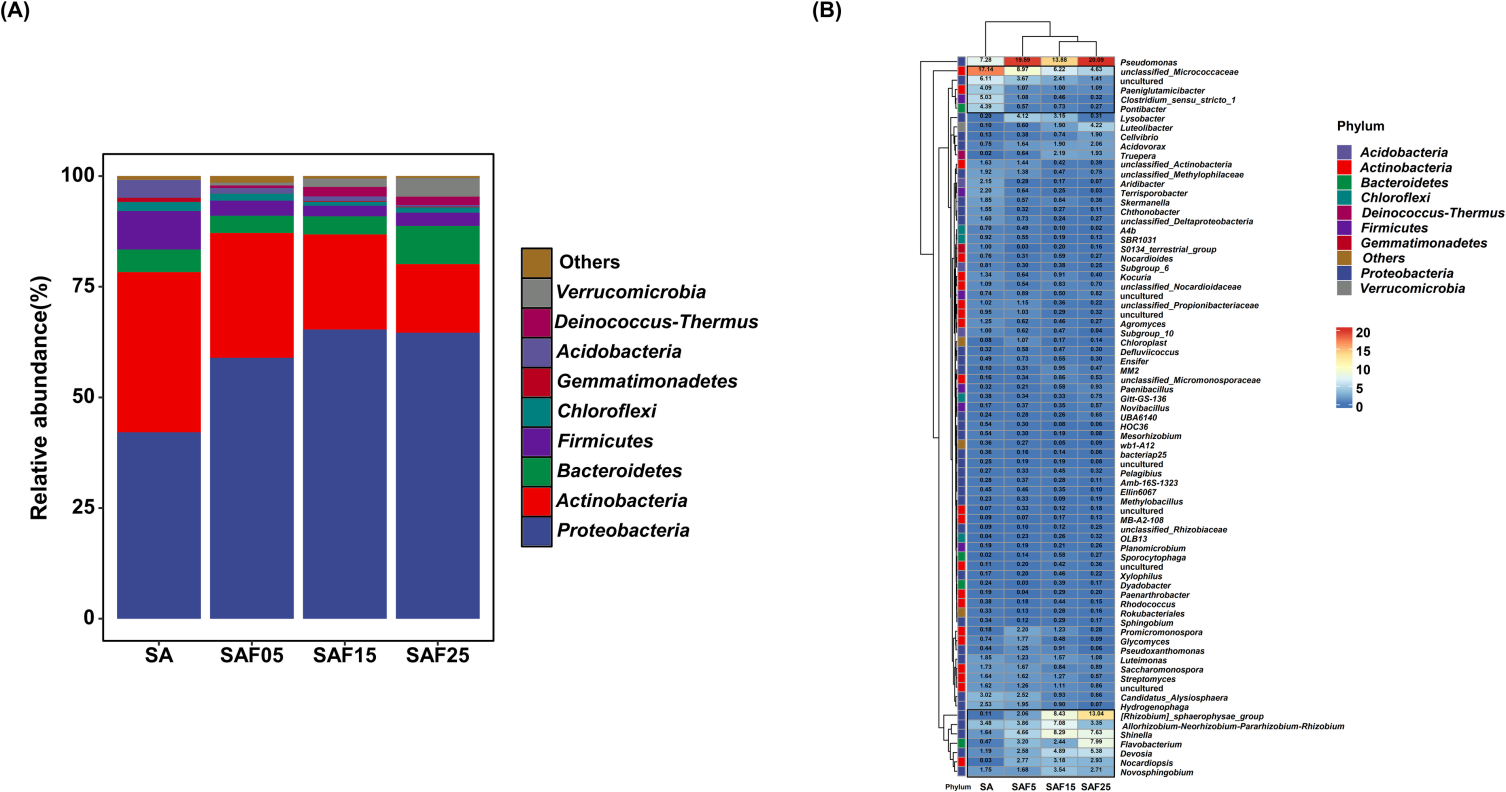
Relative abundance of core rhizosphere bacterial communities at phylum level under different compost application rates (A). The relative abundance of core rhizosphere bacterial communities at genus level was made into a clustering heatmap (B). The SA, SAF5, SAF15, and SAF25 represent different compost application rates, respectively.

Overall, 113 core ASVs were identified in alfalfa rhizosphere soils (Supplementary Table S4). The core ASVs were mainly dominated phylum by *Proteobacteria*, *Actinobacteria*, *Firmicutes*, *Bacteroidetes*, and *Acidobacteria* (Figure 4A). Compared with SA treatment, the relative abundance of *Proteobacteria* in SAF5, SAF15, and SAF25 treatments increased by 40.05, 55.24, and 53.52%, respectively. Likewise, the abundance of *Actinobacteria* displayed a decreasing trend with the increase of compost application rates. Compared with SA treatment, the relative abundance of *Bacteroidetes* in SAF5 treatment decreased by 22.94%, and decreased by 19.32% in SAF15, but increased by 69.90% in SAF25 treatment (Figure 4A). At the genus level, the relative abundances of *Allorhizobium-Neorhizobium-Pararhizobium-Rhizobium*, *shinella*, *Novosphingobium,* and *Nocardiopsis* were highest in SAF15 treatment, accounting for 7.08, 8.29, 3.54 and 3.18% of the total sequences, respectively (Figure 4B). However, the relative abundances of *unclassified_Micrococcaceae*, *uncultured*, *Clostridium_sensu_stricto_1*, and *Pontibacter* were lowest in SAF25 treatment, accounting for 4.63, 1.41, 0.32, and 0.27% of the total sequences, respectively (Figure 4B).

**Figure 5:**
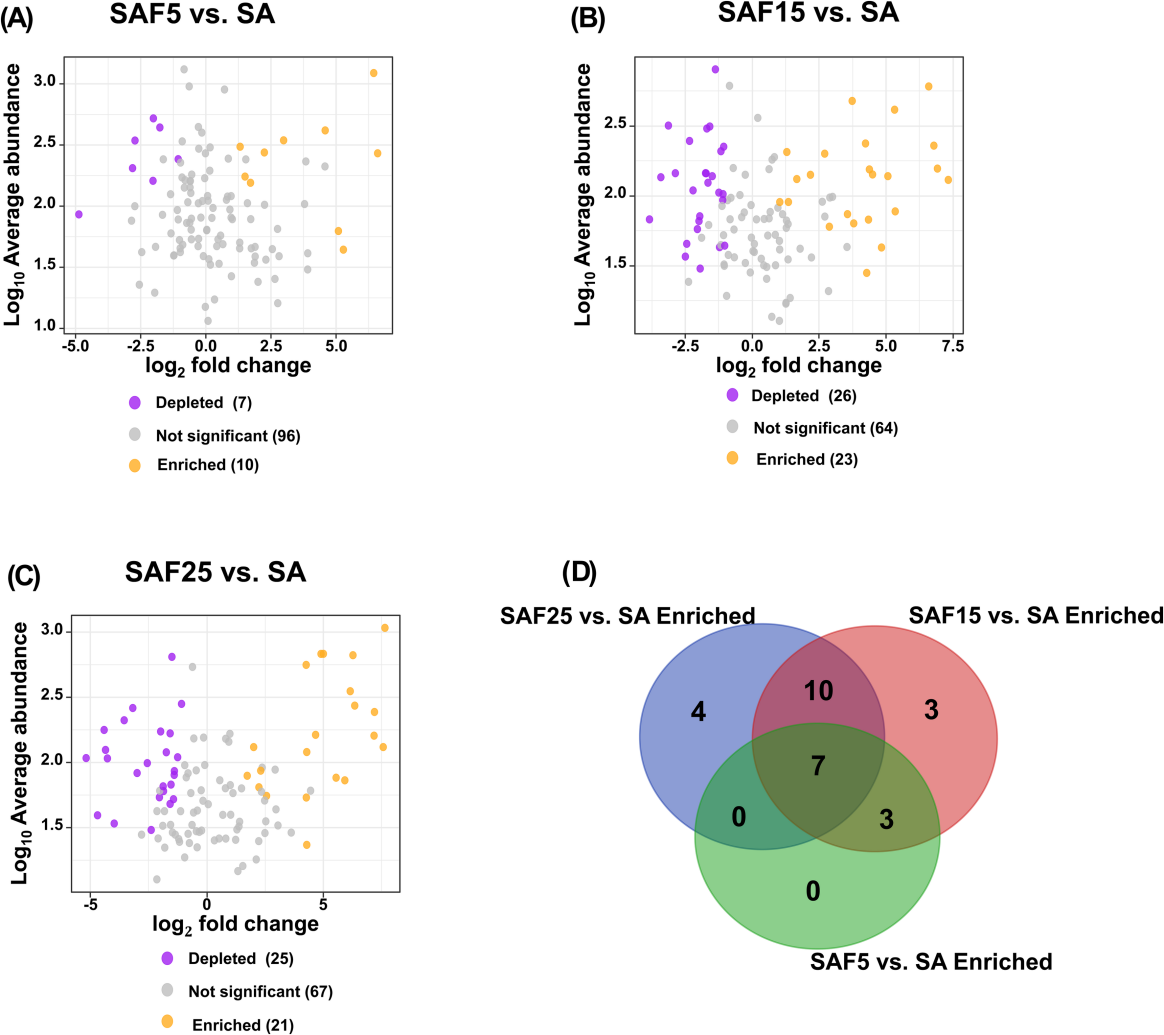
Volcano plot of the average abundance of core ASVs for different compost application rates compared to SA (A–C). Common and specific significantly enriched ASVs (D) under different compost application rates were plotted in the Venn diagram. The SA, SAF5, SAF15, and SAF25 represent different compost application rates, respectively.

Since the enriched core rhizosphere bacterial communities play an important role in plant nutrient availability (Chang et al., 2022). We performed the differential significant analysis of 113 core bacterial communities based on different compost addition treatments relative to control treatment to determine the ASVs strongly affected by different compost rates (Figure 5; Supplementary Table S5). The number of enriched ASVs in SAF15 treatment was the highest, followed by those in SAF25 treatment and SAF5 treatment (Figures 5A–C). Further, we classified the core ASVs which enriched in different comparison groups into two categories: shared significantly enriched ASVs in the SAF5 vs. SA and SAF15 vs. SA, SAF15 vs. SA and SAF25 vs. SA, SAF5 vs. SA and SAF25 vs. SA, SAF5 vs. SA and SAF15 vs. SA and SAF25 vs. SA comparisons, and specific significantly enriched ASVs in the SAF5 vs. SA, SAF15 vs. SA, SAF25 vs. SA comparisons (Figure 5D).

**Figure 6:**
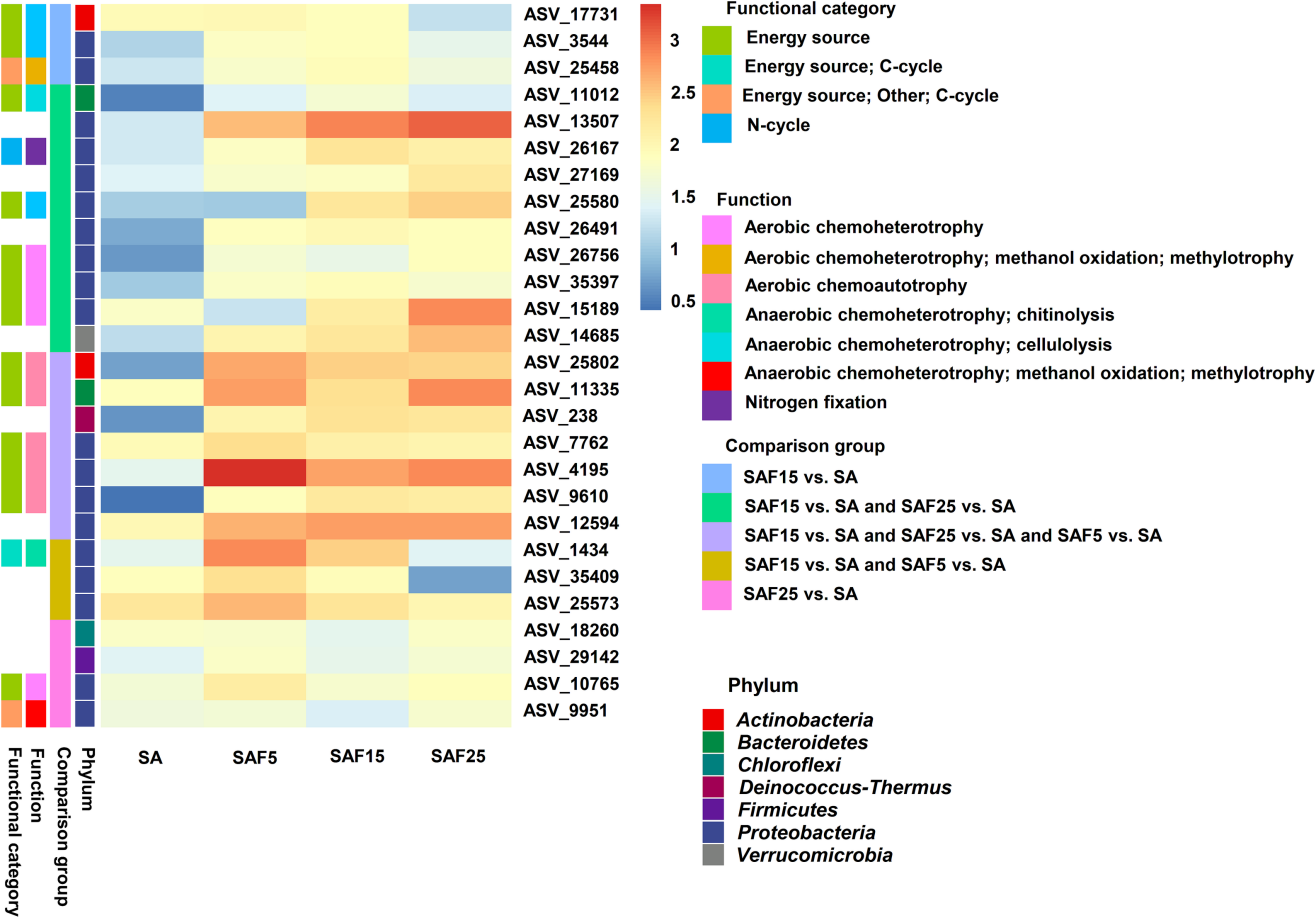
Functional classification of the common and specific significantly enriched core rhizosphere bacterial communities under different comparison groups. The shading from blue to red represents low- to high log 10 (ASV-level abundance value).

Subsequently, a total of 27 enriched core bacterial ASVs under different comparison groups were recognized by using FAPROTAX, in which only 17 (shared/specific enriched) ASVs were annotated to function, including aerobic chemoheterotrophy, nitrogen fixation, cellulolysis, anaerobic chemoheterotrophy, methanol oxidation, methylotrophy (Figure 6; Supplementary Table S6). In the SAF15 vs. SA and SAF25 vs. SA comparisons, 6 of the 10 shared significantly enriched ASVs have functional annotation information. Genus belonging to *Proteobacteria*, such as *Devosia* (ASV_25580), *Novosphingobium* (ASV_26756, ASV_35397), and *Pseudomonas* (ASV_15189), were annotated to aerobic chemoheterotrophy function. And the genus *Allorhizobium-Neorhizobium-Pararhizobium-Rhizobium* (ASV_26167) was annotated to nitrogen fixation function. In addition, genus belonging to *Bacteroidetes*, *Sporocytophaga* (ASV_11012) was annotated to anaerobic chemoheterotrophy and cellulolysis function. In the SAF15 vs.SA and SAF25 vs.SA and SAF5 vs.SA comparisons, 5 out of the 7 shared significantly enriched ASVs have functional annotation information. Genus belonging to *Proteobacteria*, such as *Devosia* (ASV_7762) and *Pseudomonas* (ASV_4195, ASV_9610), were annotated to aerobic chemoheterotrophy function. Genus belonging to *Actinobacteria*, *Nocardiopsis* (ASV_25802), and Genus belonging to *Bacteroidetes*, *Flavobacterium* (ASV_11335), were annotated to aerobic chemoheterotrophy function. In the SAF15 vs. SA and SAF5 vs. SA comparisons, 1 out of the 3 shared significantly enriched ASVs have functional annotation information. Genus belonging to *Proteobacteria*, such as *Lysobacter*, was annotated to chitinolysis. In the SAF15 vs. SA comparison, a total of 3 specific enriched ASVs have functional annotation information. Genus belonging to Proteobacteria, *Devosia* (ASV_3544) was annotated to aerobic chemoheterotrophy. Genus belonging to Actinobacteria, *Streptomyces* (ASV_17731) was annotated to aerobic chemoheterotrophy. Genus belonging to *Proteobacteria MM2* (ASV_25458), was annotated to anaerobic chemoheterotrophy, methanol oxidation，methylotrophy. In the SAF25 vs. SA comparison, 2 out of 4 specific enriched ASVs have functional annotation information. Genus belonging to *Proteobacteria*, *Acidovorax* (ASV_10765), was annotated to anaerobic chemoheterotrophy, methanol oxidation, and methylotrophy. Genus belonging to *Proteobacteria*, *UBA6140* (ASV_9951), was annotated to anaerobic chemoheterotrophy, methanol oxidation, methylotrophy.

### 3.4 The relationship of differentially enriched core ASVs to soil properties, plant traits, and their potential contribution to plant biomass

**Figure 7:**
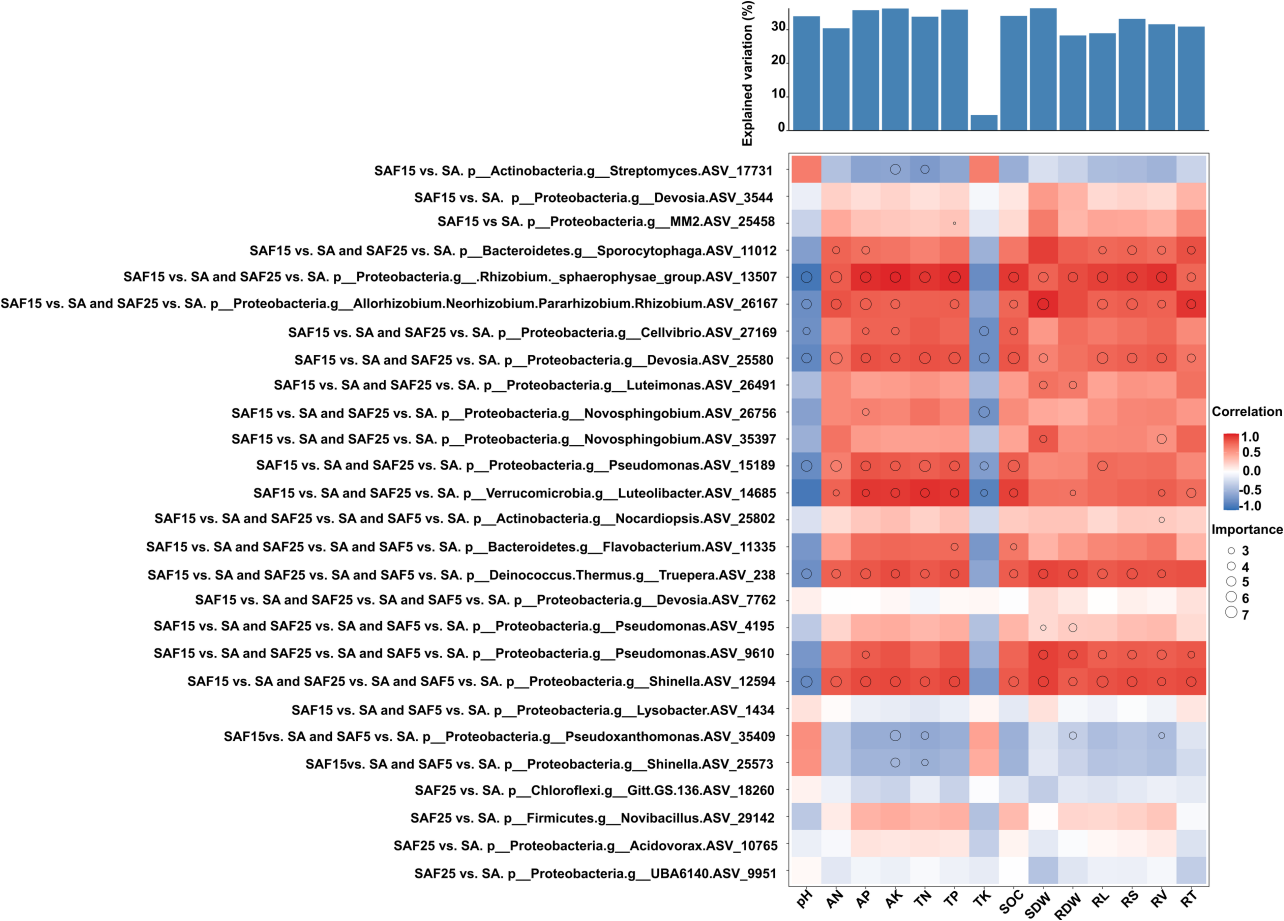
Potential contribution of different enriched core ASVs to plant root nutrient contents and plant biomass. Circle size represents the variable’s importance. The shading from blue to red represents low- to high positive Spearman correlation coefficients.

Based on the criterion of the absolute value of the correlation coefficient and important value, we analyzed the enriched core bacterial communities and their relationships with soil properties and plant traits under different compost application rates (Figure 7; Supplementary Table S7).

For example, the shared significantly enriched ASVs belonging to Phylum *Proteobacteria*, genus *Allorhizobium-Neorhizobium-Pararhizobium-Rhizobium* (ASV_26167), *Pseudomonas* (ASV_15189), *Devosia* (ASV_25580), were important and positively correlated with soil AP, AN, AK, TP and SOC. Whereas they were important and negatively correlated with soil pH. They were involved in nitrogen fixation and aerobic chemoheterotrophy function, respectively. The shared significantly enriched ASV belonging to Phylum *Bacteroidetes*, genus *Flavobacterium* (ASV_11335) was important and positively correlated with SOC and TP. The specific significantly enriched ASV belonging to Phylum *Actinobacteria*, genus *Streptomyces* (ASV_17731), was important and negatively correlated with AK and TN. It was involved in aerobic chemoheterotrophy function. The specific significantly enriched ASV belonging to Phylum *Proteobacteria*, genus MM2 (ASV_17731), was important and positively correlated with TP. It was involved in anaerobic chemoheterotrophy, methanol oxidation, and methylotrophy function.

All differently abundant active bacterial genera contributed to the variations in root nutrients and biomass. For example, the shared significantly enriched ASVs belonging to Phylum *Proteobacteria*, genus *Allorhizobium-Neorhizobium-Pararhizobium-Rhizobium* (ASV_26167), *Devosia* (ASV_25580) and *Pseudomonas* (ASV_9610), were important and positively correlated with SDW, RL, RV, RT and RS. The shared significantly enriched ASV belonging to Phylum *Bacteroidetes*, genus *Sporocytophaga* (ASV_11012) was important and positively correlated with AP, AN, RL, RV, RT, and RS. It was involved in anaerobic chemoheterotrophy and cellulolysis function. The shared significantly enriched ASV belonging to Phylum *Proteobacteria*, genus *Novosphingobium* (ASV_35397), was important and positively correlated with SDW and RV.

Further, soil chemical properties and plant traits were used to predict the contribution rate of plant biomass. The results showed that most soil chemical indexes had a greater contribution rate to plant biomass, followed by plant root traits. The contribution rates to plant biomass were SDW (36.36%), AK (36.27%) TP (35.94%), AP (35.78%), SOC (34.07%), pH (33.97%), TN (33.84%), RS (33.21%), RV (31.59%) (*p* < 0.01), respectively.

## 4 Discussion

### 4.1 Compost promotes plant growth and restores the soil environment

Composting is an effective method for recycling agricultural waste such as straw, livestock manure, and poultry manure, which can significantly reduce the environmental harm caused by the irrational use of chemical fertilizers and promote the sustainable development of agriculture (Miyamoto et al., 2023; Xu et al., 2023). Root structure reflects the ecological adaptability of plants and may increase plant survival under stress conditions (Hartle et al., 2006). In this study, compost significantly improved the accumulation of biomass of alfalfa seedlings and promoted root growth (Figure 1). It is worth noting that the plant biomass and RT in SAF15 treatment significantly increased, indicating that compost has a promoting effect on plant growth. Based on the above results, we concluded that the economic and effective ratio of compost application in saline-sodic soil may be 15%, because, under the compost application rate, it has the best effect on repairing saline-sodic soil and improving alfalfa productivity, and well below 40% of the SAF25 cost.

High-quality compost contains a large amount of organic compounds, which play a positive role in soil biological processes and can improve the physicochemical properties of the soil (Zhang et al., 2012). Recent studies have shown that compost, as a soil amendment, can significantly reduce the soil pH of saline-sodic soil and increase SOM (Xu et al., 2023), AN and AP (Siedt et al., 2021), SOC and total nitrogen (Liu et al., 2019; Lakhdar et al., 2009). Consistently, our data indicated that the soil pH significantly decreased, while the soil AP, AK, TN, TP, and SOC significantly increased with the increase of compost application rates (Table 1). We concluded that the reason was that fertilization markedly boosts the soil’s nutrient levels (carbon and nitrogen) and microbial population, stimulating an expansion of the soil carbon input pathway. The newly generated carbon, triggered by a priming effect, overly offsets the depletion of soil organic carbon, leading to a net gain of carbon in the soil (Abdalla et al., 2022). On the other hand, with an increase in the application of compost, the abundance of beneficial microorganisms increases. These microorganisms can promote the degradation and release of inorganic phosphorus through enzyme secretion, facilitate the formation of phosphorus complexes, convert insoluble inorganic phosphorus into soluble forms, making phosphorus more readily available for plant uptake, reducing soil phosphorus retention, and enhancing the effectiveness and mobility of soil phosphorus (Jin et al., 2016; Liu et al., 2019).

### 4.2 Compost regulates rhizosphere bacterial community structure

Soil biodiversity is considered to be an important indicator of the functional maintenance and sustainability of soil agroecosystems, and improvements in soil salinity are also associated with soil microbiome (Zhao et al., 2021). Soil *α*-diversity is defined as the average species diversity of different sites or habitats on a local scale, focusing on the number of species within local homogenous habitats. Different organic amendments significantly improved the αdiversity of soil bacterial communities (Mao et al., 2022). Our results demonstrated that the bacterial richness indices of Chao1, Observed_species and Faith_pd in SAF15 treatment were the highest, while the evenness index Pielou_e was lowest in SAF25 treatment (Table 2). The results showed that 15% compost had a greater effect on the rhizosphere bacterial community diversity of alfalfa, which could be attributed to bacteria are suitable to grow in the soil environment with appropriate nutrients, and suitable soil fertility can stimulate the growth of bacteria (Sharaf et al., 2021). At the same time, the introduction of species into the soil may help increase microbial diversity (Ouyang and Norton, 2020).

Microbial networks are widely used to uncover relationships among microorganisms and the stability and complexity of these relationships are essential for a comprehensive understanding of microbial ecology and the implementation of strategies for sustainable agricultural production (Deng et al., 2012). Numerous studies have indicated that microbial interactions in microbial interaction networks are influenced by fertilizer application protocols (Gu et al., 2019; Tang et al., 2023). Here, we focused on the potential effects of different compost application rates on rhizosphere bacterial symbiotic networks of alfalfa planted in saline-sodic soil. Our data revealed that compost application significantly improved the complexity of bacterial symbiotic networks, strengthened bacterial species interactions, and showed more key hubs than control. The potential interactions of rhizosphere bacterial communities in alfalfa were affected by different application thresholds of compost (Figure 3). Intriguingly, with the increase of compost application rates, the proportion of negative edges in the rhizosphere bacterial symbiosis network gradually decreased, which further indicates that the application of compost promotes the positive interaction of rhizosphere bacterial construction. Compared with other compost application thresholds, the SAF15 treatment had the highest number of total edges (Figure 3; Supplementary Table S3), which also confirmed that the bacterial community structure under the SAF15 treatment was the most complex and diverse. It is evident that organic amendments (composts) have a significant effect on the plant rhizosphere microbial community structure in saline-sodic soil, so it is not difficult to infer that the soil microbiome may play a key role in the restoration of saline soil through compost application.

### 4.3 Compost mediates recruitment of core rhizosphere bacterial communities

The core microbiota are highly interconnected taxonomic groups. Their presence or absence, as well as changes in abundance, can lead to significant alterations in microbial community structure and function, playing a crucial role in plant nutrient acquisition (Chang et al., 2022) and biological and abiotic stress adaptation (Hamonts et al., 2018). Previous studies have revealed that plant rhizosphere bacterial communities are dominated by *Proteobacteria*, *Firmicutes*, and *Actinobacteria*, and to a lesser extent by *Bacteroidetes* and *Acidobacteria* (DeAngelis et al., 2005; Weinert et al., 2011). *Proteobacterium* can degrade many large molecules and promote the circulation of carbon, nitrogen, sulfur, and other essential substances. It also plays a vital role in mitigating abiotic stress by fixing nitrogen and promoting plant growth (Bruto et al., 2014). *Actinomycetes* directly affect soil decomposition and carbon cycling processes (Upchurch et al., 2008). *Firmicutes* (Wang et al., 2020), *Acidobacteria* have been described as common inhabitants of all soils (Zhang et al., 2018; Furtak et al., 2019). In this study, with the increase of compost application rates, the relative abundance of *Proteobacteria* was gradually increased (Figure 4). Consistent with Cui’s findings, the environment with a significant increase in nutrients preferentially supports the growth of copiotrophic species after the use of organic amendments (Cui et al., 2023). Interestingly, we found the abundance of *Bacteroidetes* decreased in SAF5 and SAF15 treatments, but increased in SAF25 treatment. It was mainly due to the fact that some *Bacteroidetes* prefer a high-pH living environment, and the addition of compost reduces the pH value of the soil (Fierer et al., 2012; Ganzert et al., 2014). A higher threshold value was used in the composting process, resulting in an increase in the base value of the introduced bacteria *Flavobacterium* and *Bacteroidetes*. These bacteria can accelerate the corrosion and degradation of the composting raw straw, and their original abundance is high.

Aerobic_chemoheterotrophy is the primary pathway of carbon flow in aerobic microbial communities and is generally thought to play an important role in the circulation of organic matter in all ecosystems (McKinley and Wetzel, 1979; Kämpfer et al., 1993). It is closely related to the circulation of organic matter and the flow of energy in the soil metabolic system. In this study, the core bacteria *Pseudomonas*, *Devosia*, *Novosphingobiu*m, and *Flavobacterium* were recruited in the roots of alfalfa after compost addition. Their main ecological functions were good oxidation heterotrophic, anaerobic heterotrophic, and nitrogen fixation (Figure 6). This suggested that compost application can shape soil microbial communities and ecosystem functions (Gu et al., 2019), thereby promoting soil microorganisms’ participation in the succession of carbon cycling (Tang et al., 2023). In addition, compost also promoted the accumulation of symbiotic nitrogen-fixing bacteria in alfalfa roots. Nitrogen fixed by rhizobia in legumes can also benefit related non-legumes by transferring biofixed nitrogen directly into intercropping grains (Hayat et al., 2010). Ultimately, it can be concluded that the addition of compost in the saline soil environment changed the environmental ecological niche and promoted the evolution of soil bacterial communities.

### 4.4 Potential contributions of different enriched core ASVs to plant root nutrients contents and plant biomass

Microorganisms are vital components of soil, and plays a key role in maintaining soil biological characteristics and fertility. They improve soil quality by fixing or dissolving nutrients and adding organic matter through various biological functions and metabolic characteristics (Kumar et al., 2023). In this study, the enriched active genera in the soil rhizosphere microbiome recruited after compost application were closely related to root nutrients (Figure 5). For example, the core rhizosphere bacteria *Allorhizobium-Neorhizobium-Pararhizobium-Rhizobium*, *Devosia*, and *Pseudomonas* were important for predicting AN, AP and SOC, and were significantly positively correlated with them. Meanwhile, the core rhizosphere bacteria *Flavobacterium* was important for predicting SOC and TP (Figure 7; Supplementary Table S7). Consistently, Hu’s study observed that most functional microbial populations in soil, especially those microbial taxa involved in carbon degradation, nitrification, nitrate reduction, and organic phosphate mineralization were regulated by soil nutrient availability (Hu et al., 2024). These data suggested exploring the threshold of soil properties around roots and controlling the correlation of these properties with the core microbiome, especially with soil nutrient availability (Li et al., 2017).

Plant growth-promoting rhizobacteria (PGPR) are a type of rhizospheric bacteria that positively impact plant growth. Compared to bulk soil, they tend to thrive more abundantly in the rhizosphere (Lugtenberg and Kamilova, 2009). Root phenotype traits may affect the root and rhizosphere soil microbiome (Saleem et al., 2016). At the same time, rhizospheric microorganisms aggregate around the roots, enhancing the bioavailability of insoluble minerals, thereby increasing mineral absorption by the roots to provide nutrients to plants, and also influencing root development (Trivedi et al., 2020). In this study, the enriched core ASVs under different compost application rates, such as *Allorhizobium-Neorhizobium-Pararhizobium-Rhizobium*, *Devosia*, and *Pseudomonas*, were of great significance for predicting changes of above-ground dry weight and root morphological parameters, and showed significant positive correlations (Figures 6, 7). These enriched core ASVs were mainly involved in nitrogen fixation and aerobic chemical heterotrophic functions. This is consistent with previous findings that plants influenced and shaped beneficial microbial community structure and function through their root structure, nutrient release, and changes in soil pH, thus related to the promotion of plant growth and development (Berendsen et al., 2012; Etesami and Maheshwari, 2018). Additionally, our data showed that the enriched core ASV belonging to *Novosphingobium* was of great significance for predicting changes in SDW and RV of alfalfa seedling, and showed a significant positive correlation. This supports Fan et al.’s (2023) assertion that *Novosphingobium* is a bacterium involved in breaking down organic matter as part of the carbon cycle. The secretion of soil compounds around alfalfa roots enriches a large population of organic-degrading bacteria. Furthermore, the reduction in soil salinity around these roots further encourages the growth of these beneficial bacteria. The direct positive correlation between taxa enriched in compost-treated saline-sodic soil and specific root nutrients and plant biomass was detected in this study, which will help to identify potentially important taxa for the development of agricultural microbiome engineering solutions to improve root nutrient uptake and increase plant productivity in saline-sodic soil.

## 5 Conclusion

This study exhibited that compost provided more AK, TP, AP, SOC, and TN for the reclamation of saline-sodic soil. Compost reshaped rhizosphere microbiome, and promoted alfalfa plant growth, especially the growth potential after 15% compost application rate was the highest. The core ASVs belonging to the genus *Pseudomonas*, *Devosia*, *Novosphingobium*, and *Flavobacterium* were involved in the ecological functions of energy resources and nitrogen cycle, and played an important role in promoting the nutrient resource acquisition of alfalfa growth. The detection of an important and positive correlation among the core bacterial taxa, specific root nutrients, and plant biomass in different compost application treatments will help to identify potentially important taxa, which contribute to developing agricultural microbiome engineering solutions to improve root nutrient uptake and increase plant productivity in saline-sodic soil. In conclusion, compost can induce alfalfa roots to enrich core rhizosphere bacterial communities involved in energy source and nitrogen fixation, improve soil nutrient cycling, regulate plant growth traits, and ultimately promote plant biomass accumulation. These findings provide valuable insights for the improvement of soil quality and agricultural productivity in saline-sodic soil.

## Data Availability

The datasets presented in this study can be found in online repositories. The names of the repository/repositories and accession number(s) can be found at: https://www.ncbi.nlm.nih.gov/bioproject/PRJNA1133892.

## Footnotes

^1^https://docs.qiime2.org/2019.4/tutorials/
